# Safety Management and Safety Performance Nexus: Role of Safety Consciousness, Safety Climate, and Responsible Leadership

**DOI:** 10.3390/ijerph192013686

**Published:** 2022-10-21

**Authors:** Farida Saleem, Muhammad Imran Malik

**Affiliations:** 1 Department of Management, College of Business Administration, Prince Sultan University, Riyadh 11586, Saudi Arabia; 2 Department of Management Sciences, COMSATS University Islamabad, Attock Campus, Attock 43600, Pakistan

**Keywords:** safety performance, safety climate, safety consciousness, responsible leadership, industrial safety

## Abstract

Drawing from social system theory, social identity theory, and social exchange theory, this study examines how safety management practices are linked with employee safety performance through safety consciousness and safety climate. Furthermore, responsible leadership is introduced as a boundary condition in the safety consciousness—safety performance and safety climate—safety performance relationships. Data were collected from employees belonging to pharmaceutical firms located in different industrial zones of Lahore, Pakistan. The support is found for full mediation of safety consciousness and safety climate for the safety management and safety performance relationships. Responsible leadership moderates the safety consciousness—safety performance and safety climate—safety performance relationships so that when the safety climate is weak or the safety consciousness is low, a high level of responsible leadership enhances safety performance.

## 1. Introduction

Occupational safety and safety performance can provide competitive advantage to the firms [1] and have has become a prominent area of research in the last three decades [2,3]. The focus of this research is to identify safety-related outcomes and to provide guidance for improving health and safety in organizations. An inadequate safety management system is the root cause of the majority of industrial disasters [4]. Hence, organizations’ adoption of safety management systems is linked with their attempt to achieve performance excellence. Safety performance is one of the key factors for gaining a competitive advantage in today’s rapidly globalizing world. Effective preventive measures like safety management systems or behavior-based system approaches can help in the reduction in occupational accidents.

According to the most recent data on workplace health and safety, there are currently 2 million people who believe their illnesses were made worse by their employment, and each employee loses an average of 30 million days (1.3 days) every year because of illness or injury [5,6]. This is a result of potential carelessness on the part of the companies in maintaining safety procedures. Some businesses fail to give health and safety the priority it needs, despite the clear necessity for proactive management. This could be the result of insufficient staff resources or a lack of expertise, skills, and motivation. However, employee safety management and alertness are the keys to reducing the ratio of work-related illnesses and accidents at work. Safety management relates to the real procedures, duties, and responsibilities involved in staying safe [7,8]. Safety consciousness, on the other hand, is the awareness of risks and the vigilance for danger. It has a strong influence on the actions of an individual because of his desire to remain alive and uninjured. There is always a need to develop safety consciousness because most injuries can be traced to someone’s lack of safety consciousness [3,9]. It is a key predictor of safety outcomes that has attracted limited attention.

According to Kirwan [8], safety management is related to all practices that are associated with remaining safe, which includes actual practices, roles, and functions. Safety management is a sub-system of organizational management systems that are integrated into the organization and has a focus on controlling the hazards that can negatively affect the health and safety of employees [4]. Safety management systems not only implement policies and procedures, activities that are required to control the hazards, but also comply with the existing legislation applicable to the organization. The safety management system is an important antecedent of a safety climate [3,4] and the development of safety consciousness in employees. A safety climate is the shared perception of employees regarding the state of safety of their organization [10]. Similarly, safety consciousness is the awareness of an individual regarding the safety issues and concerns of an organization [11]; this awareness can be at both cognitive and behavioral levels [4]. Neal et al. [12], and Vinodkumar and Bhasi [4], have considered safety climate as a factor that influences the safety performance (including safety compliance and safety participation) of an organization. de Koster et al. [13] identified safety consciousness as an antecedent of the safety performance of employees in an organization. Based on social system theory (SST), social identity theory (SIT), and social exchange theory (SET), safety consciousness and safety climate are both proposed as mediators for the safety management and safety performance relationship.

A responsible leadership role in safety management and performance relationship is implicit as the majority of organizations’ central goal is to ensure the value of safety is in the minds of employees [14]. The personality, values, and choices that employees make, the people they trust, the appeals they respond to, and the way they invest their time and energy in an organization are the outcomes of the values of leadership [14,15]. Based on social identity theory (SIT) and social exchange theory (SET), responsible leadership is proposed as moderator for the safety consciousness and safety performance and safety climate and safety performance relationships.

The importance of small and medium-sized enterprises (SMEs), especially in developing countries, cannot be denied. SMEs are considered a major contributing sector to the economic development of emerging and developing economies. Similar to other developing and emerging economies, most businesses in Pakistan are SMEs [16,17]. According to one report, about 99% of economic establishments are SMEs and their GDP contribution is 40% with 26% exports from the manufacturing sector [18]. The majority of firms in the pharmaceutical industry of Pakistan are SMEs [19]. Even though safety management and its outcomes have been researched and reported from various parts of the world, there is not much evidence available from small and medium-sized pharmaceutical firms in Pakistan, where safety performance is yet to get the priority it deserves.

The aim of the current investigation is to identify the impact of safety management practices on safety performance while taking safety consciousness and safety climate as mediators and responsible leadership as the moderator in pharmaceutical firms in Pakistan. This investigation is attempting to contribute to the literature in three ways: first, by empirically investigating a comprehensive mediated moderation model of safety management and safety performance; second, by generalizing the safety management and safety performance investigations that are majorly focused on developed countries to a developing country; and last, by focusing on the pharmaceutical industry of Pakistan.

### Theoretical Framework

The theoretical foundations for this study come from three theories, namely social systems theory, social identity theory, and social exchange theory. The social system theory [20] holds social behavior as the result of the interaction of the institution’s role and expectations and individual personality and needs [21,22]. In an organization, organizational behaviors are products of interaction between the organizational factors and individual factors. The safety management practices adopted by the organizations in terms of safety-policy making, safety training, safety communication, and preventive planning lead to developing a safety climate that further boosts safety performance. Skyttner [23] stated that the emergence of anything results from the interaction of independent parts when they stop being independent and start to influence each other; therefore, it is posited that when individuals come together (the leader and employees) and develop a common sense of implementing safety in the organization it creates a safety climate in the organization, thus satisfying the social systems approach. It is noted that when individuals adopt safety practices, they try to achieve safety synergy [24], reflecting the social systems approach. Individual after individual following the safety standards combine together and make it a success.

Similarly, according to social identity theory, positive CSR perceptions (safety management, safety consciousness, safety climate, and safety performance) enhance organizational identification. This leads to the desire to maintain this positive identity and group membership, which later on translates into commitment. In safety-oriented organizations, the people feel safe and tend to retain their jobs for longer times.

**Figure 1:**
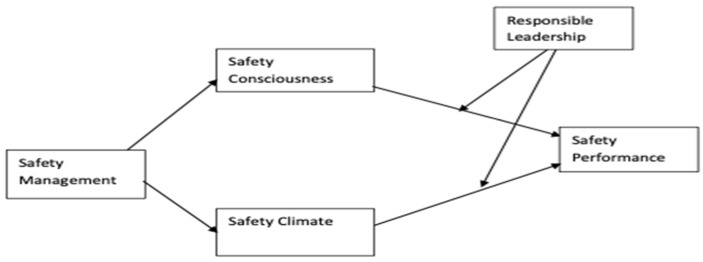
Proposed Research Framework.

The theory of social exchange [25] postulates that in any social interaction where one party acts in a manner that benefits a second party, a mutual expectation will emerge that obligates the second party to reciprocate, at some later stage, by acting in a way that benefits the first party [26]. The social exchange theory (SET) is a theory that describes relationships as result-oriented social behavior. It is based on the reciprocity of the behaviors. The social behavior in the interaction of organization and employees is used for a cost–benefit analysis to create a win–win situation [27]. In this situation of give and take, this study argues that responsible leaders, by virtue of taking care of the stakeholders [28], will ensure the formulation of effective health and safety policies and procedures, provide the necessary knowledge, skills, and abilities, provide support on such policies and procedures, communicate performance standards, and promote a safety climate. As suggested by the social exchange theory [25], responsible leaders will be fostering a trusting relationship among employees (as stakeholders) through their proactive participation in ensuring the implementation of health and safety procedures, thereby acting as role models of health and safety rules and regulations [29]. Moreover, it is noted that socially responsible behaviors such as safety behaviors cannot be implemented without the influence of the leaders [30]. Under such arguments, it is posited that when employees get something of value from their leaders, they try to give it back through their hard work and by following the practices they require from them to ensure performance targets. The employees who take training from their responsible leaders tend to stand with them and try their best to practice safety at the workplace, thus enforcing the social exchange. The proposed research framework is presented in Figure 1.

## 2. Literature Review

### 2.1. Safety Management and Safety Consciousness

According to Barling et al. [11], safety consciousness consists of two components: the cognitive component and the behavioral component. This indicates that the idea goes beyond only being aware of safety risks and that taking necessary action is important too [31,32]. Furthermore, in the organizations, the modeling of behaviors depends upon demands put forth by the top managers. The managers emphasize the importance of health and safety policies and procedures and will inspire subordinates to ponder safety, hence increasing their safety consciousness [33]. Similarly, safety management requires clear communication of the health and safety policies. This requires the provision of training to the employees to enhance their subordinates’ consciousness. Studies suggest that consciousness is an important predictor of safety behaviors.

The main goal of safety management is to prevent workplace injuries, illnesses, and deaths, as well as the suffering and financial hardships of the organizations [34]. The recommended practices use a proactive approach to managing workplace safety and health, instead of using the reactive ones, i.e., problems are addressed only after a worker is injured or becomes sick. These recommended practices recognize that finding and fixing hazards before they cause injury or illness is a far more effective approach [35].

The rate of accidents can be minimized through safety management and consciousness. This relationship forms a pattern that affects the well-being of all workers. The factor of luck may distort the pattern, but over a long period of time the pattern remains unchanged [36]. The employees are required to perform in a safer way that would not harm themselves and their co-workers [37]. Common causes of error may include time pressure, mental pressure, fatigue, being new to the task, distractions, and overconfidence.

The safety management practices enforced through safety policies, plans, procedures, training, and frequent safety communication enable people to avoid accidents [13,38]. The push to follow the safety practices from the managers adds to the consciousness. This consciousness as mindfulness brings positive results for individuals and organizations. Safety-conscious workers are more likely to notice potential risks, make unbiased judgments, and control their unsafe or risky behaviors [39,40]. Bahari [41] conducted a safety-related study and found that the employees’ safety can be improved by employees’ understanding of safety, knowledge about safety, and the skills necessary to ensure safety. Moreover, the management’s attitude and actions toward safety were found to be crucial in improving organizational safety.

Accidents at work generally occur because of deficient knowledge or training, deficient supervision, and deficient procedures to carry out task safety [42]. The organizations can prevent the dangers via a safety policy implementation across the organization by setting safety objectives. This enables employees to achieve the set safety objectives, which directly means facing low risks and damage [43]. At the same time, preventive planning is a key to ensuring safety in organizations [44].

An effective way to promote safety management is safety communication. Pandit et al. [45] found safety communication to be an effective way of promoting safety management in the organization and poor safety communication can lead to disasters. It is also noted that when not only the managers but also the employees do not communicate frequently about the hazards involved in their work and possible preventive measures to be taken, this leads them to unexpected injuries. Safety training is equally important and enables employees to learn the relevant knowledge, skills, and abilities to tackle possible dangers [9]. The social systems theory posits that when components of a system combine and work in the same direction, they achieve a synergetic safety working (see Figure 1). Therefore, the hypothesis developed is:

**Hypothesis** **1.** *There is a positive impact of safety management on safety consciousness*.

### 2.2. Safety Management and Safety Climate

Management concern for safety develops the safety climate. The safety climate is the perceptions and attitudes of the organization’s workforce about surface features of the culture of safety in the organization at a given point in time [46]. Safety management is the adoption of practices to reduce errors, which fosters a safe climate in the organization. A better safety climate in an organization is associated with committing fewer errors and better outcomes.

Anticipated benefits would stem from the ability of organizations (use of safety practices) with strong safety climates to cultivate behaviors that enhance collective learning by addressing unproductive beliefs and attitudes about errors, their cause and cure [47].

According to Mearns et al. [48], the organizations that want safe operations have to ensure a safe climate. Research has focused on supervisors as role models for instilling safety awareness and supporting safe behavior [48]. Involvement of the workforce in safety-decision-making has also received attention [49]. These things require a consideration of the safety philosophy of upper management and the safety management system of the organization. Organizations with lower accident rates were characterized by the presence of upper managers who were personally involved in safety activities, the prioritization of safety in meetings and in decisions concerning work practices, and the thorough investigation of incidents [48,50]. The accumulation of the safety practices and compelling employees to follow the safety measures while at work makes a safety climate in the organization.

Guo et al. [51] noted that the climate can be developed through management emphasizing safety practices. A safety climate consists of social support, management safety commitment, knowledge of safety, and pressure of production. Management’s commitment to safety has a direct relationship with social support [52]. Hence, management should establish clear policies on safety and safety issues that encourage people to follow safety standards. The present study posits that the managers with safety concerns will ensure the formulation of effective health and safety policies and procedures, provide necessary training on such policies and procedures, communicate performance standards, and promote a safety culture [53].

The safety climate is the shared perceptions of employees about the importance of safety within the organization. This is developed when the individual parts work together and develop a common sense of safety in the organization to make a system, as per social systems theory (see Figure 1). In the light of such arguments the hypothesis developed is:

**Hypothesis** **2.** *There is a positive impact of safety management on safety climate*.

### 2.3. Safety Climate and Safety Performance

Griffin and Neal [54] argued that employees’ perceptions of the policies, procedures, and practices relating to safety comprise the safety climate. The safety climate acts as a frame of reference for the behavior and attitudes of individuals and groups of employees, and it is argued that it will also affect their accident involvement. The employees with more favorable safety perceptions (indicating a positive safety climate) are less likely to engage in unsafe acts [55]. Safety performance is defined by Neal et al. [12] as the level of safety compliance and safety participation. Safety compliance means “adhering to safety procedures and carrying out work in a safe manner”, and safety participation means “helping co-workers, promoting the safety program within the workplace, demonstrating initiative and putting effort into improving safety in the workplace” (p. 101).

Humans play an important role in the occurrence of workplace accidents, but the safety climate can achieve excellence in prevention [56]. At the individual level, the safety climate is concerned with employees’ understanding of safety stimuli such as practices, procedures, and policies in the workplace. The safety climate, in fact, serves as a benchmark for directing and guiding suitable and adaptive safety behavior [57].

Guo et al. [51] believe that if individuals have favorable perceptions of safety, they are less likely to act unsafely on site. As a result, accident rates are likely to decline. As such, a safety climate can cause a profound change in employees’ behavior and mentality, leading to true safety implementation, thus enhancing safety performance. Borgheipour et al. [52] found a positive result for a safety climate influencing safety performance. The safety climate inculcates danger-avoiding practices and improves safety performance. The safety climate encourages employees to learn safety practices, thus fostering safety performance.

Jafari et al. [58] worked on the development of the safety climate scale and found 10 dimensions, namely management commitment, workers’ empowerment, communication, blame culture, safety training, job satisfaction, an interpersonal relationship, supervision, continuous improvement, and a reward system, to be effective in making the safety climate. Management commitment to safety and safety training make people capable of better safety performance. Eskandari et al. [59] developed a scale for measuring safety performance. They considered three factors for their examination, such as the organizational factors, the environmental factors, and the individual factors. They found organizational factors had the highest contribution toward safety performance. A safe climate is rightly considered an organizational factor that encompasses a common understanding of safety among employees.

A safety climate, that is, the common perceptions and attitudes of the employees about ensuring safety practices in the organization at a given point in time [46], leads to minimizing errors at work. This further leads to efficient working and low waste-age of resources, thus ensuring safety performance in the organization. Clarke [60] stated that the assumption underlying the link between an organizational safety climate and the accident rate is that climate provides guidance on suitable organizational behavior, so that a more positive climate encourages safe behaviors through organizational rewards (e.g., recognition and feedback for making safety suggestions), while a more negative safety climate reinforces unsafe behaviors by removing incentives to improve safety (e.g., prioritizing production over safety), which, in turn, are related to the occurrence of workplace accidents.

The social identity theory posits that positive corporate responsibility perceptions like providing safety training to the stakeholders, i.e., employees, having frequent communication, and so forth, leads to safety performance. The safety ensured in work leads to organizational identification. People tend to work in the organizations characterized by safety [61]. The hypothesis developed is:

**Hypothesis** **3.** *There is a positive impact of a safety climate on safety performance*.

### 2.4. Safety Consciousness and Safety Performance

The employees’ knowledge about safety standards encourages them to ensure safety performance. Worker engagement in safety may systematically act to reduce the probability of human errors from occurring by making workers more involved with and aware of their tasks/surroundings and associated risks, as well as the error traps that could be present. Thus, increased levels of worker engagement in safety activities could possibly be related to increased safety performance as measured by standard safety outcomes, i.e., accident rates [38].

Knowledge about safety standards and practices leads to enhanced levels of cognitive engagement [62]. The employees display focus, attention, and concentration on the safety aspects of the job. By displaying safety behavior, the employees working in the organization are known to be safety-minded people. Safety mindedness leads to leading others by example. They are the ones who are fond of continuous learning. They respond to feedback quickly and have strong communication skills. Safety-conscious people try not to harm others and make people learn safety practices by adopting safety organizational citizenship behaviors [63]. A positive safety climate is developed when the employees have a perception of management safety values and commitment to safety [48].

Safety consciousness refers to an “individual’s own awareness of safety issues” [11]. This awareness works on both a cognitive and a behavioral level. Cognitively, safety consciousness means being mentally aware of safety in your work and knowing what behaviors foster operational safety. Behaviorally, safety consciousness enacts the behaviors that foster operational safety. Safety consciousness can be separated from the safety climate in a manner that safety consciousness is about the safety of oneself, whereas a safety climate is about the safety of the whole workplace in the organization. We argue that the extent to which the individuals are aware of the safety hazards and are aware of possibly avoiding them indicates whether they are in a better position to minimize the accidents, i.e., the safety performance. Safety performance is the extent to which companies are able to prevent accidents and errors.

Kelloway et al. [64] argued that if anything could reduce the chances of accidents, it would be employees’ awareness of issues that threaten safety, their knowledge of how to prevent them, and their behaviors oriented toward preventing them (i.e., safety consciousness). Safety consciousness comes from the segments of the organization working together. Individuals, when seeing one another following the safety principles, tend to adopt safe work practices, which thus resembles the social systems theory. In light of the above arguments, the hypothesis developed is:

**Hypothesis** **4.** *There is a positive impact of safety consciousness on safety performance*.

### 2.5. Responsible Leadership as Moderator for Safety Consciousness and Safety Performance

Clarke [60] noted that “there is very limited understanding of the impact of leadership styles on safety outcomes” (p. 1175). Except for a transformational leadership style, empowering leadership, and safety leadership, which drew more research attention, leadership styles have not been adequately investigated in this context [65,66,67]. Many argue that leaders are the prime drivers of high-reliability organizations (i.e., [68]). For example, top management is often responsible for the implementation of safety-enhancing systems and the development of a safety-oriented culture. When the responsible leaders weigh different stakeholder claims before deciding, it helps in building trust and people feel free to share their safety problems and seek solutions. This enhances safety consciousness and ensures safety performance. Rare evidence is available in the literature explaining responsible leadership with relation to safety outcomes. However, it is possible that when the leaders act as role models, as per social exchange theory, the people adopt the same behaviors as their role models [69]; this boosts safety compliance and performance.

Abbas et al. [70] argued that employees are the critical stakeholders of organizations and are responsible for protecting the organizational environment via their safety mindfulness and interpersonal interaction. The safety of mindfulness and interactions are shaped by the responsible leader’s powerful forces of protection, acquisition, connection, and understanding [71]. In the presence of reinforcement from the responsible leaders, this mindfulness further leads to better safety performance.

**Hypothesis** **5.** *Responsible leadership significantly moderates the relationship between safety consciousness and safety performance*.

### 2.6. Responsible Leadership as Moderator for Safety Climate and Safety Performance

Leaders demonstrate normatively appropriate conduct through personal actions and interpersonal relationships and promote such conduct to subordinates through two-way communication, reinforcement, and decision-making [72]. Rare evidence is available for responsible leaders influencing the safety outcomes. However, this mechanism can be explained as responsible leadership being characterized as involving stakeholders in decision-making and looking after their demands. Moreover, he has an idea of the consequences of his decisions on the stakeholders. This enables the employees to get involved in the safety procedures and they conduct periodic checks on the execution of the prevention plans. Furthermore, they participate in evaluating the risks. This premise is based on the social exchange theory. The positive exchanges taking place between the leader and the employees lead to compliance as a matter of showing gratitude. The hypothesis developed is:

**Hypothesis** **6.** *Responsible leadership significantly moderates the relationship between safety climate and safety performance*.

## 3. Methodology

The population of the current study includes small and medium-sized pharmaceutical firms located in industrial zones of Lahore, Pakistan. A list of 100 small and medium-sized pharmaceutical firms operating in the industrial zone near Lahore, Pakistan was compiled. The criteria of the National SME Policy 2007 of Pakistan, “an enterprise with an employment size up to 250, capital of Rs. 25 million and annual sales up to Rs. 250 million”, was used for defining an SME. Out of 100 firms, 37 agreed to participate in the study; hence, these 37 firms were contacted for data collection. CEO, plant manager, production manager, quality assurance manager, quality control manager, pharmacists, technical staff, and assistant managers were contacted via Google Forms, which was shared either through email or WhatsApp. A total of 209 fully completed self-report surveys were received and used for data analysis.

### 3.1. Data Collection

Self-administered survey forms were used for data collection. The purpose of the survey was to analyze the behavior of respondents toward the safety management practices, safety climate, level of safety consciousness, and safety performance of their firm and responsible leadership. A non-probability, convenience sampling technique was employed for respondent selection and data collection.

### 3.2. Instrumentation

For the measurement of safety management, an 18-item scale was adopted from Beatriz Fernández-Muñiz et al. [73]. The safety management scale has four subcategories, namely safety policy, training in safety, communication in prevention issues, and preventive planning. For the measurement of safety consciousness, a seven-item scale was adopted from Westaby and Lee [9]. Item samples are “I always take extra time to do things safely” and “People think of me as being an extremely safety-minded”. A safety climate scale was adopted from Beatriz Fernández-Muñiz et al. [73] with seven items. Sample items are “Periodic checks conducted on execution of prevention plans and compliance level of regulations” and “Accidents and incidents reported, investigated, analyzed, and recorded”. The variable safety performances were measured with the help of an eight-item scale developed and used by Beatriz Fernández-Muñiz et al. [74]. Similarly, a scale of responsible leadership with five items was adopted from Voegtlin [75]. Sample items are “My direct supervisor demonstrates awareness of the relevant stakeholder claims” and “My direct supervisor considers the consequences of decisions for the affected stakeholders”. The survey instrument/questionnaire is attached in Appendix A.

## 4. Data Analysis

### 4.1. Descriptive Analysis

Majority of data collected were from males. Out of 209 responses, 128 were males, while the remaining 81 were females. Similarly, the majority of respondents were of the age group between 30–39 and were from lower- to middle-level management categories; 14% were holding undergraduate degrees, 67% had master’s degrees, and the remaining 18% had higher-level degrees.

### 4.2. Common Method Variance

Self-reported data raise the issue of the potential effect of common method variance (CMV) [76]. Prior to hypothesis testing, CMV was tested using Harman’s one-factor test by loading all items into a single factor. The results revealed that 33% of the variance is explained by the single factor that is below the recommended threshold value of 50%. The result revealed that the data are free from CMV.

### 4.3. Scale Validation

Confirmatory factor analysis (CFA), also known as the measurement model, was used as an analytical strategy for the validation of the scale. CFA was conducted using AMOS 17. SM 18, and SP 7 was removed at this stage as it was not successfully loaded into its latent construct. The results of CFA provided acceptable model-fit indices and are presented in Table 1.

### 4.4. Statistical Assumptions

The statistical assumptions including normality, reliability, and validity of the collected data were checked before hypothesis testing.

#### 4.4.1. Normality Analysis

Univariate normality can be accessed through skewness and kurtosis indices, which should lie between the absolute value of 3 and 10, respectively [77]. The skewness values for the current data lies between −1.888 and 0.210, while kurtosis values were between −0.928 and 3.05, hence showing univariate normality in the dataset.

#### 4.4.2. Reliability Analysis

Internal consistency and reliability of the dataset was checked using both Cronbatch’s alpha values and composite reliability. The alpha values were calculated using SPSS 20, while composite reliability measures were obtained through CFA output. The overall scale provided the alpha of 0.911 while the alpha values were between 0.903 and 0.973 and composite reliability values were between 0.907–0.975 for each latent construct. The Cronbatch’s alpha and composite reliability values for each latent construct presented in the model are given in Table 1.

#### 4.4.3. Validity Analysis

Convergent validity can be achieved by getting the loadings of observed variables on their respective latent constructs significant (*p* < 0.001) and the squared multiple correlation value of each observed variable greater than 0.5. The validity analysis results indicated that the dataset was valid for further analysis. The values of squared multiple correlation are presented in Table 2.

Similarly, the discriminant validity for the dataset was evaluated using the criteria presented by Fornell and Larker [78], where the shared variance of any construct should not be greater than the average variance extracted (AVE). The AVE value for every variable was greater than the shared variance of all variables, hence indicating a discriminant validity of the data.

### 4.5. Hypotheses Testing

All proposed hypotheses were tested using PROCESS Macro by Hayes [79]. PROCESS Macro was preferred over other analytical techniques because of its robustness and bootstrapping approach. The PROCESS Macro provides biased corrected 95% CI and can simultaneously analyze the moderation and mediation effect for complex models.

We have used an incremental approach to test our hypotheses, where at the first step we assessed two mediation models by taking safety consciousness and safety climate as mediators. After that, two moderation models taking safety consciousness and safety climate, respectively, were analyzed. Finally, the full mediation moderation model was assessed. In total, five models using PROCESS Macro have been analyzed.

### 4.6. Mediation Model

To test the first set of proposed hypotheses, PROCESS Macro (extension in SPSS) by Hayes [79] Model No. 4 was used. The results identified that SM has insignificant direct effect (B = −0.0641; *p* > 0.10) on SP. However, the indirect effect through SC is significant (B = 0.086; *p* < 0.10), hence identifying full mediation. Similarly, for safety climate as mediator, the SM has insignificant direct (B = −0.047; *p* > 0.10) and significant indirect effects (B = 0.069; *p* < 0.10) through safety climate, providing support for full mediation. The results of PROCESS Model 4 are presented in Table 3 and Table 4.

### 4.7. Moderation Analysis

PROCESS Macro (extension in SPSS) by Hayes [79] Model No. 1 was used to test the proposed moderation hypotheses for responsible leadership. PROCESS Macro by Hayes [79] was preferred over simple regression analysis using interaction term and structural equation modeling because of its robustness. PROCESS Macro uses a bootstrapping approach with biased corrected at 95% confidence intervals that calculates the Johnson-Neyman outputs for the interaction term. The variables that define product term were first mean centered. Conditioning values at mean and ±1SD and Johnson-Neyman outputs for the interaction graph were also calculated. We have used a separate PROCESS Model No. 1 for safety consciousness and safety climate. The result of PROCESS Model 1 are presented in Table 5.

**Figure 2:**
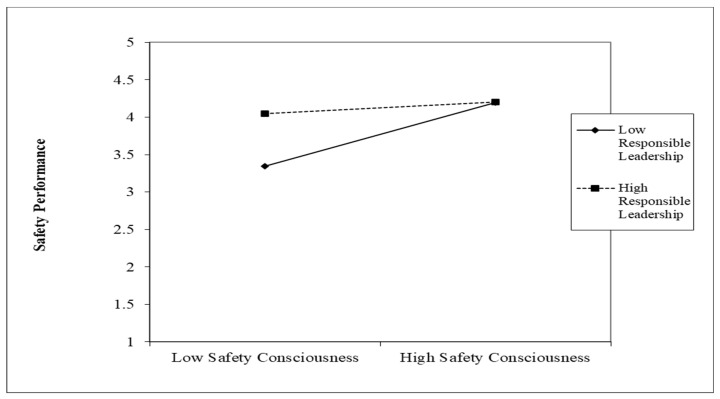
Interaction plot for responsible leadership and safety consciousness.

**Figure 3:**
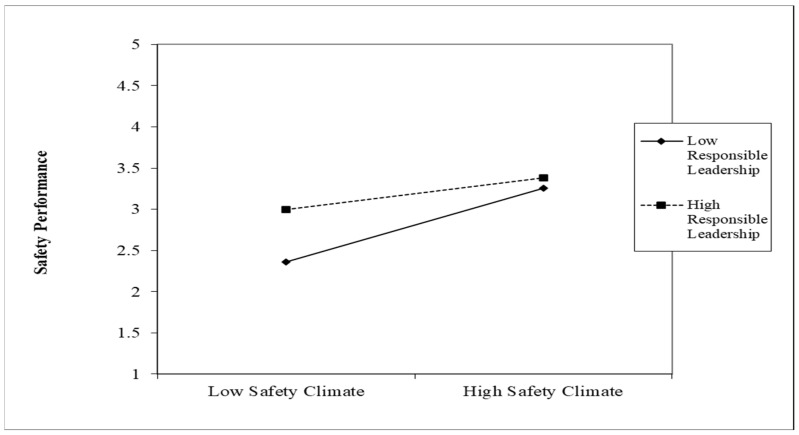
Interaction plot for responsible leadership and safety climate.

The results identified that the interaction terms for both SC (B = −0.163 *; *p* < 0.01) and SCL (B = −0.117 **; *p* < 0.05) were significant and there was no zero in the lower and upper bound of the 95% confidence interval. Interaction graphs for low and high (Mean ± SD) values of SC and RL and SCL and RL were plotted. The interaction graph of the SC and RL relationship (shown in Figure 2) suggests that RL significantly enhances the relationship between safety consciousness and safety performance when safety consciousness is low. The role of RL as moderator is significant at low levels of safety consciousness, and it becomes insignificant when safety consciousness is high. Similarly, for safety climate, in the interaction graph of the RL and SCL relationship (shown in Figure 3), RL is significant at low levels of safety climate. The slope test shows that the presence of RL enhances the positive relationship of SP and SC and SP and SCL when SC and SCL are low.

### 4.8. Mediated Moderation Analysis

Finally, to test mediation and moderation simultaneously, we have used PROCESS Macro Model No. 14 with 5000 bootstraps sampling and 95% biased corrected confidence intervals. We have run PROCESS Model No. 14 twice, one with safety consciousness as a mediator and one with safety climate as mediator. The results of moderated mediation analysis identified that there is a significant indirect effect of SM on SP through both SC and SCL. Out of the two proposed mediators, only safety consciousness has a significant index of mediated moderation (index = −0.457; LB: −0.1053; UB: −0.0069). Similarly, the conditional indirect effects (indirect effects in presence of moderators) of SM on SP were significant. The results of PROCESS Model No. 14 are presented in Table 6.

## 5. Discussion

Due to the considerable human and monetary costs associated with workplace accidents, it is important to study various factors that help reduce mishaps [80]. Various factors are examined to test the framework of this study. The results show a positive relationship between safety management and safety consciousness (Hypothesis 1) in the workplace. This study supports the findings of the earlier studies [81,82]. The employees are required to perform in a safer way that would not harm themselves and their co-workers [37]. This consciousness can be enhanced through developing a plan for work, reporting all the possible hazards, safety training, reporting all the hazards that actually occurred during the work, accepting the responsibility for any misconduct and taking measures to avoid them in the future, and teaching ourselves and our colleagues about preventing the accidents [38].

The safety standards and procedures enforced by the organization keep employees in touch with the use of safety procedures and can avoid dangerous situations [9]. Safety management can be made effective through safety policy, as a part of safety climate (Hypothesis 2). The policy helps coordinate the other HR policies to ensure employees’ well-being. The written policies show the management’s concern for safety and this written document reminds the employees about the safety standards to be implemented while at work. The written documents also provide the procedures to follow to avoid any possible health hazards [13]. The policies available lead to continuous improvements in the workplace and save all from the dangers. Another important component of safety management is the priority of providing training to the employees to avoid any uneven situation at the workplace. The avoidance of dangerous situations and compelling others to be safe by themselves and keep others safe becomes mandatory while working, thus boosting safety consciousness [41].

The managers who provide safety training as a part of a safety climate, as a priority to learn safety measures, provide an opportunity to make a safe competitive environment that is safety performance (Hypothesis 3). People try to learn as much as they can to avoid accidents, thus adding to their safety consciousness [41]. The organizations must communicate about the risks associated with work. Frequent communications can help in two ways. First, the employees become aware of the hazards associated with their work, and, second, if anything goes wrong it will be well-communicated in time to reduce the damage as much as possible [45]. Better safety management frequently transmits the principles and rules of action. This makes people mindful of safety. Preventive planning is another way to add timely emergency plans that can reduce the possible level of danger at the workplace. Involving employees in making preventive plans can enable people to think of their safety, thus making people conscious of their safety. In short, policies, training, communication, and preventive planning collectively motivate employees to remain conscious of their workplace safety for positive outcomes [44] (Hypothesis 4).

The results of this study found a positive impact of safety management on the safety climate. Safety training, communication, preventive planning, and policy-making are important for developing a common sense of safety in the organization [41]. A written declaration available to and signed by all workers reflects management’s concern for safety. This puts compulsion on the employees to develop a safe climate. Moreover, the safety policy puts emphasis on commitment to continuous improvement. This leads to conducting periodic checks on the execution of prevention plans [81].

The continuous and periodic training of employees enables them to apply their safety knowledge, skills, and abilities in a manner that saves themselves and their co-workers [9]. This also helps to keep procedures in place and to check the achievement of the objectives set. Similarly, the instruction manuals or work procedures elaborated can help employees prevent accidents. The frequent communication for transmitting the principles and rules of action leads to collective awareness [45]. Moreover, the written circulars and meetings inform workers about risks associated with their work and how to prevent possible accidents. Similarly, the written circulars and meetings force employees to conduct systematic inspections periodically. This ensures the effective functioning of the whole system.

Organizations that focus on emergency plans by practicing responsible leadership can protect employees from harm. Effective organizations always have emergency plans in place. The prevention plans are based on an assessment of the risk and employees are empowered to know the possible degree of risk. They keep themselves aware of the risks and try their best to be alert while assuming risky tasks [43] (Hypothesis 6).

The results show a positive impact of the safety climate on safety performance, i.e., consistent with the results [52,58,59]. The improvement in the safety climate means reducing potential safety threats. The employees conduct periodic checks as a result of the frequent communication and training provided to them. This enhances the compliance levels and ensures safety in the workplace [81]. The timetables available for the safety checks support compliance. The periodic checks of the procedures and equipment to use keep track of the personal protection equipment and keep the inventory up to date. That further strengthens the safety performance. The predetermined plans enable employees to improve working conditions by following the standard operating procedures and minimizing the errors involved. The minimized errors save time and other resources from wastage [47].

The results confirm the positivity in the relationship of these two variables, such as the findings of Lee et al. [82] and Wong et al. [81]. The higher the knowledge of the safety standards, the greater the application of the safety standards can be ensured. Moreover, one who is convinced about the use of safety procedures can compel himself and others to remain safety-minded [63]. At times it becomes difficult for the employees to use the safety equipment, but the policies and rules enforce the adoption of safety measures. The employees who remain involved in the evaluation of the safety risks tend to show discomfort when they see other people acting dangerously [44].

In the safety-enforced environment, the employees are compelled to make sure that other people do the things that are safe and healthy. Participation in setting the safety objectives and improving safety practices also gives a sense of ensuring collective safety at the workplace by inculcating safety-mindedness [52]. Compliance with the safety standards encourages employees to avoid dangerous situations. Participation of employees in the risk evaluations and safety inspections, and in making suggestions about improving the safety situation at the workplace, compel them to do the safest possible things as the best strategy [38].

Results found that responsible leadership is significant when the safety consciousness and safety performance relationship is low. The leaders play important roles in shaping safety behaviors (Hypothesis 5). As per the social exchange theory, socially responsible behaviors such as safety behaviors cannot be implemented without the influence of the leaders [30]. The leader’s concern to take care of the stakeholders makes a stronger bond to keep closely in touch with the employees. The employees keep in touch with the leaders and share their concerns and problems about safety. The leader guides them and involves them in decision-making and setting the safety parameters. This involvement and the knowledge of the safety practices enhance the consciousness of employees and they become capable of avoiding accidents.

The results show that responsible leadership is a moderator for the safety climate and safety performance relationship (Hypothesis 6). The social exchange theory supports the leader’s involvement in shaping the safety climate. The leaders who make people learn and develop a common sense of safety in the organization help people learn and retain the knowledge. This further leads to ensuring safety performance. Based on social identity theory and social exchange theory, this study aims to investigate the mechanisms underlying the responsible leadership, safety consciousness, and safety performance relationship.

Responsibility is one of the essential components of effective leadership in the field of organizational management [83]. Due to a lack of leadership accountability in today’s accident-prone climate, businesses are experiencing a crisis of low trust. Lack of trust by employees make them pay less attention to the safety instructions given by their leaders, which leads to mishaps and a loss of health and safety. As a result, executives need to act more responsibly toward employees and other stakeholders. The socially responsible leaders are more effective and have greater impact on the organizations than other leadership styles [84]. As per social identity theory, the people are inclined to categorize themselves and others into social groups and establish a positive self-concept by identifying with groups that enhance their self-esteem.

Furthermore, individuals tend to boost their self-image by identifying with organizations recognized for their social engagement and responsibility [85], which subsequently motivates employees to strive for organizational safety objectives.

Additionally, people tend to feel better about themselves when they associate with organizations that are known for their social responsibility, such as with a safety climate [85]. This inspires employees to work toward the achievement of organizational safety objectives.

Furthermore, social exchange theory posits that individuals’ voluntary actions are motivated by the returns they expect from others [25]; the responsible leaders take care of the stakeholders and in return the employees take care of the leaders and try to oblige them by following the safety standards, thus showing safety performance.

## 6. Conclusions

Three theories are supported through this examination: the social identity theory, the social systems theory, and the social exchange theory. Safety consciousness and safety climate are necessary for showing safety performance. Moreover, responsible leadership is the key to achieve better safety performance in the organization when the safety climate is weak or safety consciousness is low.

## 7. Implications

The following practical implications can be drawn from this investigation for the managers and policymakers. Prevention of accidents is only possible through inculcating safety consciousness, a safety climate, and safety performance in the organizations with responsible leaders who can enforce and implement safety standards in the organization through policy making, planning, training, and communication.

Safety management practices have a direct effect on the safety consciousness of the employees. Safety management can foster safety consciousness by means of policy-making, training, communication, and preventive planning that possibly protect the organizations from accidents and disasters. Employees with safety consciousness have a greater chance to avoid accidents and can remain healthy to perform without errors. The higher the level of consciousness, the greater will be the safety practices applied while doing work. Merely the presence of safety equipment or kits is not necessary, but the employees must have the tendency to remain safe and use the kits available to prevent dangers associated with their work.

The direct influence of management commitment on safety compliance can be considered as a result of the individual wisdom of the employees, earned from the overall interest shown by the management toward the safety of their employees, to protect themselves from accidents. Safety performance can become a priority for the employees by having the management’s strong concern for safety. The role of responsible leadership is significant for developing safety consciousness, a safety climate, and safety performance of employees. The traits of responsible leadership should be developed in other top managers to prevent accidents.

## 8. Limitations

Like other research studies, this study has a few limitations. First, only a single sector/organization was selected for the study, which provided a limited sample size. Therefore, one should be cautious in generalizing the results of this study to other industries/sectors/organizations. Second, the companies with poor safety practices may have been reluctant to provide an original response to the questionnaire; therefore, there may be an influence from selection bias. Third, the survey itself was cross-sectional. A future longitudinal study could provide stronger support for causal relationships between safety climate and safety outcomes vis-a-vis the responsible leadership style used for the study.

## Figures and Tables

**Table 1:** Results of confirmatory factor analysis (CFA).

Construct/Variable	βeta	Alpha	CR	AVE
Safety Management		0.973	0.975	0.740
SM1	0.775			
SM2	0.863			
SM3	0.866			
SM4	0.829			
SM5	0.831			
SM6	0.877			
SM7	0.833			
SM8	0.833			
SM9	0.778			
SM10	0.762			
SM11	0.787			
SM12	0.825			
SM13	0.853			
SM14	0.846			
SM15	0.862			
SM16	0.830			
SM17	0.780			
SM18				
Safety Consciousness		0.948	0.949	0.728
SC1	0.766			
SC2	0.843			
SC3	0.889			
SC4	0.858			
SC5	0.868			
SC6	0.895			
SC7	0.847			
Safety Climate		0.959	0.960	0.774
SCL1	0.830			
SCL2	0.867			
SCL3	0.889			
SCL4	0.889			
SCL5	0.906			
SCL6	0.897			
SCL7	0.880			
Responsible Leadership		0.903	0.907	0.662
RL1	0.744			
RL2	0.841			
RL3	0.886			
RL4	0.834			
RL5	0.753			
Safety Performance		0.948	0.949	0.728
SP1	0.848			
SP2	0.864			
SP3	0.862			
SP4	0.831			
SP5	0.859			
SP6	0.819			
SP8	0.887			
Goodness of fit indices				
χ^2^ = 1482; d.f. = 845; χ^2^/d.f. = 1.75; *p* < 0.001; CFI = 0.93; GFI = 0.76; AGFI = 0.73; RMR = 0.05; RMSEA = 0.06				

β: standardized coefficient; alpha: Cronbath’s alpha; CR: composite reliability; AVE: average variance extracted.

**Table 2:** Descriptive statistics and correlations.

	Variable	No of Items	Mean	s.d.	SM	SC	SCL	RL	SP
1	SM	17	3.84	0.88	0.740				
2	SC	7	3.15	1.04	0.241 ** (0.058)	0.728			
3	SCL	7	3.44	1.06	0.159 * (0.025)	0.800 ** (0.640)	0.774		
4	RL	5	2.93	1.01	0.005 (0.0002)	0.233 ** (0.054)	0.181 ** (0.033)	0.662	
5	SP	7	3.91	0.89	0.024 (0.0005)	0.343 ** (0.1180	0.431 ** (0.186)	−0.286 ** (0.082)	0.728

* correlation significant at 0.05. ** correlation significant at 0.01 shared variance are in parenthesis. AVE is on diagonal.

**Table 3:** Five thousand bootstrap results for direct and indirect effects. PROCESS Model 4 (safety consciousness as mediator).

Path	Estimate		SE	
SM→SP (Direct Effect)	−0.0641		0.07	
SM→SC	0.285 *		0.08	
SC→SP	0.310 *		0.06	
Standardized Direct and Indirect Effects using 5000 Bootstrap 95% CI				
Path	Effect	SE	LL 95% CI	UL 95% CI
Direct Effect	−0.641	0.07	−0.199	0.071
Indirect Effect (SM→SC→SP)	0.086 *	0.04	0.025	0.164

SM: safety management; SP: safety performance: SC: safety consciousness; * *p* < 0.10.

**Table 4:** Five thousand bootstrap results for direct and indirect effects. PROCESS Model 4 (safety climate as mediator).

Path	Estimate		SE	
SM→SP (Direct Effect)	−0.047		0.06	
SM→SCL	0.192 *		0.08	
SCL→SP	0.371 *		0.05	
Standardized Direct and Indirect Effects using 5000 Bootstrap 95% CI				
Path	Effect	SE	LL 95% CI	UL 95% CI
Direct Effect	−0.047	0.06	−0.175	0.081
Indirect Effect (SM→SCL→SP)	0.069 *	0.04	0.066	0.144

SM: safety management; SP: safety performance: SCL: safety climate; * *p* < 0.10.

**Table 5:** Five thousand bootstrap results for PROCESS Model No. 1, simple moderation analysis.

	DV: SP				DV: SP			
	Estimate	SE	LL 95% CI	UL 95% CI	Estimate	SE	LL 95% CI	UL 95% CI
SC	0.241 *	0.055	0.131	0.351				
RL	0.174 *	0.057	0.061	0.287				
SC*RL	−0.163 *	0.057	−0.265	−0.061				
SCL					0.302 *	0.053	0.196	0.407
RL					0.186 *	0.054	0.078	0.293
SCL*RL					−0.117 **	0.048	−0.212	−0.022
Model Fit								
F-value	17.22 *				23.02 *			
R2	0.20				0.25			
R2 Change	0.04 *				0.02 **			

SM: safety management; SP: safety performance: SCL: safety climate; SC: safety consciousness; RL: Responsible Leadership * *p* < 0.01, ** *p* < 0.05.

**Table 6:** Five thousand bootstrap results for PROCESS Model No. 14, mediated moderation analysis.

Path	Estimate		SE	
SM→SP (conditional direct Effect)	−0.025		0.06	
SM→SC	0.285 *		0.08	
SC→SP	0.247 *		0.06	
RL→SP	0.1730 *		0.06	
RL*SC→SP	−0.161 *		0.05	
SM→SP (conditional direct Effect)	−0.016		0.06	
SM→SCL	0.3045 *		0.05	
SCL→SP	0.192 **		0.08	
RL→SP	0.1856 *		0.05	
RL*SCL→SP	−0.167 **		0.07	
Conditional indirect effects of X on Y in presence of moderator using 5000 bootstrap 95% CI				
Path	Effect	SE	LL 95% CI	UL 95% CI
SA→SC→SP	0.4096 *	0.07	0.263	0.556
SA→SCL→SP	0.4214 *	0.06	0.296	0.547

SM: safety management; SP: safety performance: SCL: safety climate; SC: safety consciousness RL: Responsible Leadership, * *p* < 0.01, ** *p* < 0.05.

## Data Availability

The data will be available on request from the corresponding author.

## Appendix A. Survey Questionnaire

Safety consciousness adopted from and Westaby Lee [9]

1. I always take extra time to do things safely.
2. People think of me as being an extremely safety-minded person.
3. I always avoid dangerous situations.
4. I take a lot of extra time to do something safely even if it slows my performance.
5. I often find myself making sure that other people do things that are safe and healthy.
6. I get upset when I see other people acting dangerously.
7. Doing the safest possible thing is always the best thing.

Safety compliance and performance adopted from Beatriz Fernández-Muñiz et al. [74]

1. I always comply with the safety standards and procedures.
2. I am convinced about the importance of the safety procedures.
3. I use the personal protection equipment even if it is uncomfortable.
4. I participate in setting objectives and drawing up plans to improve safety.
5. I participate in evaluating risk.
6. I participate in the safety inspections.
7. I make suggestions about how to improve the working conditions.
8. I frequently discuss safety problems with their superiors.

Safety climate adopted from Beatriz Fernández-Muñiz et al. [73]

1. Periodic checks conducted on execution of prevention plans and compliance level of regulations.
2. Standards or pre-determined plans and actions are compared, evaluating implementation and efficacy in order to identify corrective action.
3. Procedures in place (reports, periodic statistics) to check achievement of objectives allocated to managers.
4. Systematic inspections conducted periodically to ensure effective functioning of whole system.
5. Accidents and incidents reported, investigated, analyzed, and recorded.
6. Firm’s accident rates regularly compared with those of other organizations from same sector using similar production processes.
7. Firm’s techniques and management practices regularly compared with those of other organizations from all sectors, to obtain new ideas about management of similar problems.

Safety management adopted from Beatriz Fernández-Muñiz et al. [73]

1. Safety policy:
  (1) Firm coordinates its health and safety policies with other HR policies to ensure commitment and well-being of workers.
  (2) Written declaration is available to all workers reflecting management’s concern for safety, principles of action and objectives to achieve.
  (3) Safety policy contains commitment to continuous improvement, attempting to improve objectives already achieved.
2. Training in safety:
  (1) Worker given sufficient training period when entering firm, changing jobs or using new technique.
  (2) Training actions continuous and periodic, integrated in formally established training plan.
  (3) Training plan decided jointly with workers or their representatives.
  (4) Firm helps workers to train in-house (leave, grants).
  (5) Instruction manuals or work procedures elaborated to aid in preventive action.
3. Communication in prevention issues:
  (1) There is a fluent communication embodied in periodic and frequent meetings, campaigns or oral presentations to transmit principles and rules of action.
  (2) Information systems made available to affected workers prior to modifications and changes in production processes, job positions or expected investments.
  (3) Written circulars elaborated on and meetings organized to inform workers about risks associated with their work and how to prevent accidents.
4. Preventive planning:
  (1) Prevention plans formulated setting measures to take on basis of information provided by risks assessment in all job positions.
  (2) Standards of action or work procedures elaborated on basis of risk evaluation.
  (3) Prevention plans circulated among all workers.
  (4) Firm has elaborated emergency plan for serious risks or catastrophes.
  (5) Firm has implemented its emergency plan.
  (6) All workers informed about emergency plan.
  (7) Periodic simulations carried out to check efficacy of emergency plan.

Responsible leadership adopted from Voegtlin (2011) [75]

1. My direct supervisor demonstrates awareness of the relevant stakeholder claims.
2. My direct supervisor considers the consequences of decisions for the affected stakeholders.
3. My direct supervisor involves the affected stakeholders in the decision-making process.
4. My direct supervisor weighs different stakeholder claims before making a decision.
5. My direct supervisor tries to achieve a consensus among the affected stakeholders.
